# RURAL FAMILY CAREGIVING: PROMOTING SELF-CARE AND WELL-BEING OF RURAL FEMALE CAREGIVERS OF FRAIL OLDER FAMILY MEMBERS

**DOI:** 10.1093/geroni/igae098.1831

**Published:** 2024-12-31

**Authors:** Nasreen Lalani, Bhagyashree Katare, Sampada Wagle, Siqi Yang

**Affiliations:** Purdue University, West Lafayette, Indiana, United States; Purdue University, West Lafayette, Indiana, United States; Purdue University, West Lafayette, Indiana, United States; Purdue University, West Lafayette, Indiana, United States

## Abstract

Caring for frail older adults in rural areas is often more challenging due to several disparities. Informal caregivers, mostly females carry the higher burden of caregiving for their older family members. They experience physical, psychological, and economic strains causing poor well-being and caregiver outcomes. Our study aims to examine the self-care practices of rural female caregivers and determine socio-ecological-cultural factors contributing to their resilience and subjective well-being. Using a sequential mixed-method design, 160 Qualtrics-based surveys were collected followed by 20 in-depth interviews from female rural caregivers of older adults from twelve rural counties in the north-central region of the Midwest, US. Multiple linear regressions were done to measure the associations among the study variables whereas thematic analysis was done to analyze the qualitative data. Preliminary findings suggest higher involvement in self-care practices was associated with greater subjective well-being (β=0.575, p< 0.001), and reduced depression (β=0.406, p=0.017) among the rural female caregivers. Social support positively influenced subjective well-being (β=0.588, p=0.010). Initial themes generated from the qualitative data included increased burnout, mental exhaustion, loneliness, and no time for self-care. Meaning-making and faith were the motivating factors toward caregiving roles, self-care, and well-being. Caregiver support and well-being is our social responsibility to improve social, mental, and economic outcomes and prevent rural disparities. Community-based caregiver support programs and policies should be designed using gender equity and cultural lens to improve the overall health, coping, and well-being of caregivers from a rural context.

